# Acute intestinal obstruction secondary to left paraduodenal hernia: a case report and literature review

**DOI:** 10.1186/1749-7922-8-5

**Published:** 2013-01-16

**Authors:** Waleed Al-Khyatt, Smeer Aggarwal, James Birchall, Tomothy E Rowlands

**Affiliations:** 1 Division of General Surgery and Radiology, Royal Derby Hospital, Uttoxetter Road, Derby DE22 3DT, UK; 2 Division of Surgery, School of Graduate Entry Medicine and Health, University of Nottingham, Royal Derby Hospital, Uttoxeter Road, Derby, DE22 3DT, UK

## Abstract

**Introduction:**

An internal hernia is a protrusion of bowel through a normal or abnormal orifice in the peritoneum or mesentery. Although they are considered as a rare cause of intestinal obstruction, paraduodenal hernias are the most common type of congenital hernias.

**Methods:**

A literature search using PubMed was performed to identify all published cases of left paraduodenal hernia (LPDH).

**Results:**

In Literature search between 1980 and 2012 using PubMed revealed only 44 case reports before the present one. Median age was 47 years (range 18 – 82 years). Nearly 50% reported previous mild symptoms. Two-third of patients required emergency surgery in form of laparotomy or laparoscopic repair. Reduction of hernia contents with widening or suture repair of the hernia orifice were the most common standards in surgical management of LPDH.

**Conclusion:**

Intestinal obstruction secondary to internal hernias is a rare presentation. High index of suspicion and preoperative imaging are essential to make an early diagnosis in order to improve outcome.

## Introduction

Internal hernia is, either congenital or acquired, a rare cause of small-bowel obstruction, with a reported incidence of less than 2% [1]. Paraduodenal hernias, which are a type of internal hernia, occur due to malrotation of midgut and form a potential space near the ligament of Treitz [2]. Incidental finding at laparotomy or on imaging is the most common presentation of these hernias [3]. Nevertheless, Paraduodenal hernias can lead to bowel obstruction, ischemia, and perforation with a high mortality. Left paraduodenal hernia (LPDH) is the most common types of congenital hernias and accounts for more than 40% of all cases [4]. Clinical diagnosis of LPDH is a real challenge as symptoms are entirely nonspecific. Therefore, a timely and correct diagnosis with a rapid diagnostic tool is mandatory [5]. In this review we discuss the clinical presentation and management of small bowel obstruction secondary to LPDH.

## Case presentation

**Figure 1:**
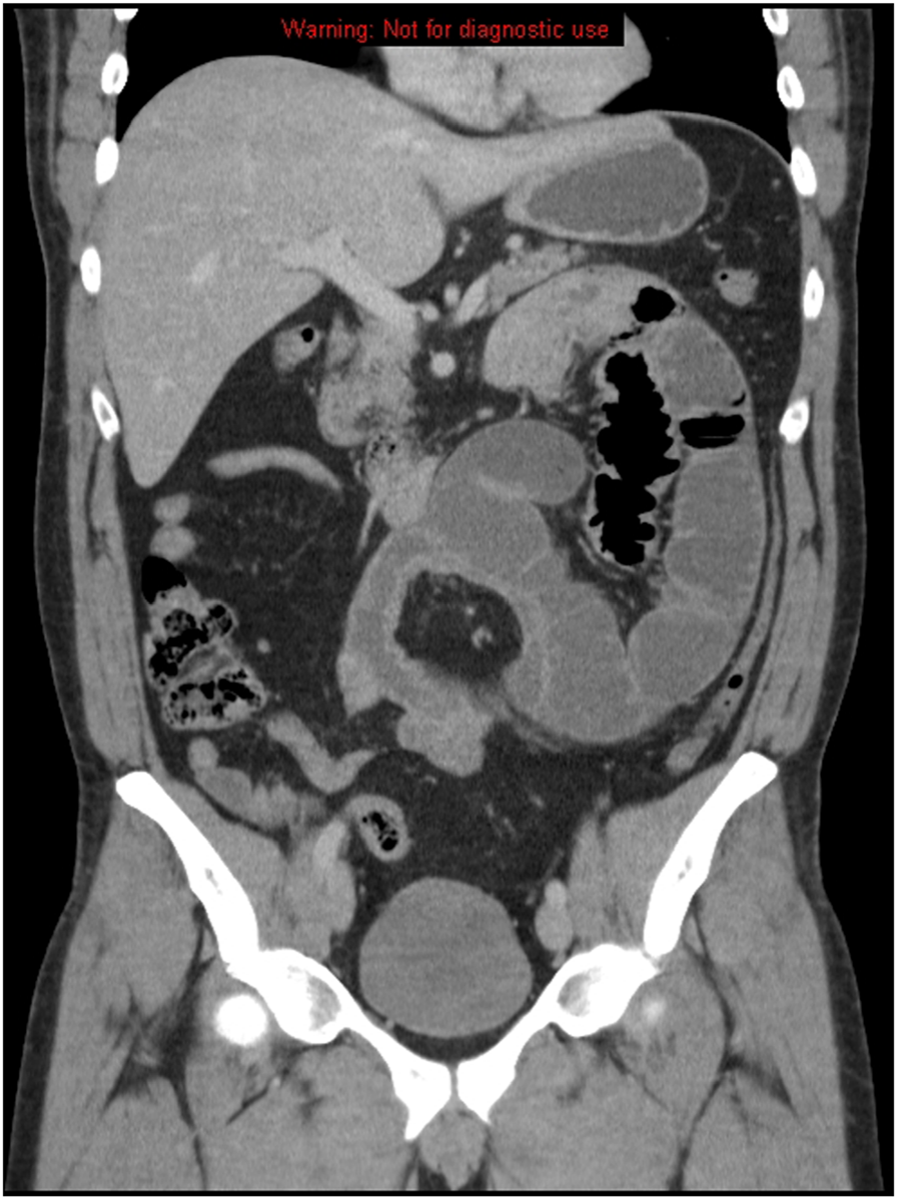
Axial enhanced CT demonstrates a cluster of dilated jejunal loops located in the Landzert´s fossa.

**Figure 2:**
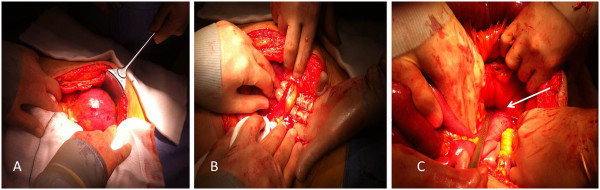
**A)Operative finding of hernia sac in the fossa of Landzert containing small bowel loops.B**) Abnormal congenital band (ligament of Treitz) containing inferior mesenteric vein. **C**) A potential space in the large bowel mesentery (arrow) with hernia sac was laid opened.

A 47 –year-old Caucasian male admitted with increasing severe colicky abdominal pain and bile stained vomiting of 2 days duration. He had no previous significant past medical or surgical history. He also denied any history of weight loss, or recent changes in his bowel habit. However, He described at least 4 previous episodes of upper abdominal distension and vomiting with spontaneous resolution over the previous 2 years. On examination, the patient appeared in moderate pain with normal vital signs. Abdominal examination revealed abdominal distension with a tender mass in the left upper quadrant. Laboratory studies were essentially normal. An urgent abdominal CT scan confirmed the diagnosis of small bowel obstruction secondary to what looked like a hernia into the left paraduodenal fossa (fossa of Landzert) (Figure 1). At laparotomy, a hernia sac of 25 cm in diameter arising from a defect just to the left of the fourth part of the duodenum was found, consistent with a LPDH (Figure 2A). The intestinal loops were herniated through that congenital defect and were not spontaneously reducible. A band containing the inferior mesenteric vein was deemed necessary to divide at the time in order to widen the orifice of the defect and to retrieve the dilated small bowel from the hernia sac (Figure 2B). The hernia sac was excised completely down to the base at the mesentery of large bowel (Figure 2C). The patient had uneventful postoperative recovery and discharged home 5 days later. At 8 weeks post-surgery, he was back to full normal activities with a well-healed laparotomy scar.

## Discussion

Internal herniation of the small bowel is a relatively rare cause of intestinal obstruction and accounts for less than 2% of all causes [1]. Among all congenital hernias, paraduodenal hernias are the most common type with an overall incidence of approximately 50% of all internal hernias [1,4,6]. LPDH (hernia of Lanzert) is about three times more common than the right counterpart (Waldayer’s hernia) [7]. LPDH arises from the fossa of Landzert, a congenital defect which presents in approximately 2% of the population, located to the left of the fourth part of the duodenum, posterior to the inferior mesenteric vein and left branches of the middle colic artery (Figure 2A) [2,8,9]. Small bowel loops (usually jujenum) prolapse posteroinferiorly through the fossa to the left of the fourth part of the duodenum into the left portion of the transverse mesocolon. Hence, the herniated small bowel loops may become trapped within this mesenteric sac (Figure 2C) [4,10].

Literature search between 1980 and 2012 using PubMed revealed only 44 case reports before the present one [2,5,11-49] (Table 1). Median age at presentation was 47 (range of 18–82 years old) with male to female ratio of 3:1. In this review, patients often presented with symptoms and signs of typical of internal hernias complicated by bowel obstruction, strangulation, and/or necrosis. Besides, 43% of patients reported a prior history of recurring abdominal pain with symptoms. Only three cases presented with a palpable mass in the left upper quadrant at time of presentation.

**Table 1:** Reported cases of left paraduodenal hernia

**Author,year**	**Age(years)**	**Gender**	**Chronic symptoms**	**Small bowel obstruction**	**Left paraduodenal hernia confirmed on imaging**	**Emergency/elective surgery**	**Laparotomy**	**Laparoscopic**
Chatterjee et al., 2012 [11]	55	Male	-	Yes	-	Emergency	Yes	-
Bhatti et al., 2012 [12]	18	Female	-	Yes	-	Emergency	Yes	-
Akbulut et al., 2012 [13]	42	Male	-	Yes	-	Emergency	Yes	-
Hussein et al. 2012 [14]	59	Female	-	Yes	Yes	Emergency	-	Yes
Fernandez-Ray et al. 2011 [15]	39	Male	-	Yes	Yes	Emergency	Yes	-
Downes et al., 2010 [16]	47	Male	Yes	-	-	Emergency	Yes	-
Parmar et al.,2010 [17]	38	Male	Yes	-	-	Elective	-	Yes
Khalaileh et al., 2010 [5]	53	Female	-	Yes	Yes	Emergency	-	yes
Yun et al., 2010 [18]	28	Male	-	-	Yes	Emergency	Yes	-
Uchiyam et al., 2009 [19]	80	Female	Yes	-	-	Elective	-	Yes
Poultsides et al., 2009 [20]	67	Female	-	Yes	-	Emergency	-	Yes
Kuzinkovas et al., 2008 [21]	59	Male	-	-	-	Elective	Yes	-
Peters et al., 2008 [22]	76	Male	-	Yes	Yes	Emergency	Yes	-
Jeong et al., 2008 [23]	52	Male	-	Yes	-	Emergency	-	Yes
Jeong et al., 2008 [23]	58	Female	-	Yes	-	Emergency	-	Yes
Palanivelu et al., 2008 [24]	-	Male	-	-	Yes	Elective	-	Yes
Palanivelu et al., 2008 [24]	-	Male	-	Yes	Yes	Emergency	-	Yes
Palanivelu et al., 2008 [24]	-	Female	-	Yes	Yes	Elective	-	Yes
Shoji et al., 2007 [25]	60	Male	-	-	-	Emergency	-	Yes
Papaziogas et al., 2007 [26]	35	Female	-	Yes	-	Emergency	Yes	-
Moon et al., 2006 [27]	18	Male	-	Yes	-	Emergency	-	Yes
Brehm et al. 2006 [28]	54	Female	Yes	-	Yes	Emergency	Yes	-
Thoma et al., 2006 [29]	72	Female	Yes	-	-	Elective	Yes	-
Cingi et al., 2006 [30]	30	Male	Yes	-	-	Emergency	Yes	-
Kurachi et al., 2006 [31]	47	Female	-	Yes	-	Emergency	Yes	-
Huang et al., 2005 [32]	24	Male	Yes	Yes	-	Emergency	Yes	-
Ovali et al., 2005 [33]	52	Female	Yes	-	Yes	Refused surgery	-	-
Fukunaga et al., 2004 [34]	51	Male	Yes	Yes	Yes	Emergency	-	Yes
Rollins et al., 2004 [35]	21	Male	Yes	-	Yes	Elective	-	Yes
Patti et al., 2004 [36]	46	Male	Yes	-	-	Elective	Yes	-
Catalano et al., 2004 [37]	82	Male	-	Yes	Yes	Emergency	Yes	-
Goodney et al., 2004 [38]	75	Male	Yes	-	-	Elective	Yes	-
Tong et al., 2002 [39]	30	Male	Yes	-	-	Elective	Yes	-
Nishida et al., 2001 [40]	47	Male	Yes	-	Yes	Elective	Yes	Yes
Patil et al., 1999 [41]	29	Female	-	-	Yes	Emergency	Yes	-
Schaffler et al., 1999 [42]	26	Male	Yes	-	Yes	Elective	Yes	-
Uematsu et al., 1998 [43]	44	Male	Yes	-	-	Elective	-	Yes
Hirasaki et al., 1998 [44]	28	Female	Yes	-	Yes	Elective	Yes	-
Mcdonagh et al., 1996 [45]	52	Male	-	Yes	-	Emergency	Yes	-
Suchato et al., 1996 [46]	40	Male	-	Yes	-	Emergency	Yes	-
Suchato et al., 1996 [46]	52	Male	Yes	-	-	Emergency	Yes	-
Warshauer et al., 1992 [47]	42	Female	Yes	-	Yes	Elective	Yes	-
Toit et al., 1986 [48]	22	Male	-	Yes	-	Emergency	Yes	-
Tireli et al., 1982 [49]	18	Male	-	Yes	-	Emergency	Yes	-

Radiological diagnosis of LPDH prior to surgery was achieved in 43% of patients. On CT scan, typical appearance of LPDH is an encapsulated sac containing clusters of dilated small bowel loops at or above the ligament of Treitz with a mass like effect compressing the posterior gastric wall and distal part of the duodenum. Besides, there is engorgement and crowding of the mesenteric vessels with frequent right displacement of the main mesenteric trunk and depression of the transverse colon (Figure 1).

Once a LPDH is identified, operative treatment is necessary, as patients with a LPDH have a 50% lifetime risk of developing small bowel obstruction with a 20–50% mortality rate for acute presentations [6,8]. In this review, 28 patients (67%) underwent emergency surgery. Of those 43 patients, 15 patients had laparoscopic repair of LPDH. Surgical intervention included reduction of the herniated small bowel loops and closure of the hernia orifice with non-absorbable sutures or a mesh [5,24]. A different possibility was to widen the hernia orifice to prevent future incarceration of bowel loops [5]. Often, there is a close anatomical relationship between the inferior mesenteric vein which bound the hernia anteriorly, and the hernia orifice [5,24]. Therefore, division of the inferior mesenteric vessels at the neck of the sac may be necessary, as in this case, when the incarcerated bowel could not be reduced easily from the hernia [24].

## Conclusion

Left paraduodenal fossa hernia is a relatively a rare cause of small bowel obstruction. In young patients with recurrent small bowel obstruction with no previous surgical history, it is crucial to consider internal hernias in the differential diagnosis. Furthermore, a timely and correct diagnosis is together with prompt surgical intervention is essential for achieving patient’s cure and prevents future complications.

## Consent

Written informed consent was obtained from the patient for publication of this case report and accompanying images. A copy of the written consent is available for review by the Editor-in-Chief of this journal.

## Competing interests

The authors declare that they have no competing interests.

## Authors’ contributions

WAK, SA, JB, and TER prepared the manuscript. TER outlined the manuscript’s layout and supervised the work. All authors read and approved the final manuscript.
